# A Case Report of Generalized Pustular Psoriasis Associated With IgA Nephropathy

**DOI:** 10.7759/cureus.10090

**Published:** 2020-08-28

**Authors:** Artsiom Klimko, Georgiana A Toma, Laura Ion, Ana Maria Mehedinti, Iuliana Andreiana

**Affiliations:** 1 Division of Physiology and Neuroscience, "Carol Davila" University of Medicine and Pharmacy, Bucharest, ROU; 2 General Medicine, University of Medicine Pharmacy Science and Technology Targu Mures, Targu Mures, ROU; 3 Department of Nephrology and Dialysis, "Dr. Carol Davila" Teaching Hospital of Nephrology, Bucharest, ROU; 4 Department of Internal Medicine and Nephrology, "Carol Davila" University of Medicine and Pharmacy, Bucharest, ROU

**Keywords:** psoriasis, iga nephropathy, glomerulonephritis, nephrotic, nephritic, psoriatic nephropathy

## Abstract

Psoriasis vulgaris is a complex immune-mediated disorder that manifests as a chronic skin disorder, characterized by well-circumscribed inflammatory, erythematous plaques. In this case report, we present a patient with generalized pustular psoriasis (GPP) who presented to the nephrology department with rapidly progressive decline in renal function. The diagnosis of GPP was made a month ago, secondary to a coagulase-negative staphylococcal superinfection. Intrinsically, this introduced a diagnostic challenge as the presumed diagnosis of immunoglobulin A (IgA) nephropathy had to be distinguished from IgA-dominant infection-related glomerulonephritis. We further discuss the current evidence and immunohistological profiles of IgA nephropathy in psoriasis and detail the evolution of renal function of our patient over 25 months after he presented to our department.

## Introduction

Psoriasis vulgaris is a complex immune-mediated disorder that manifests as a chronic skin disorder, characterized by well-circumscribed inflammatory, erythematous plaques. The prevalence rates of this disease vary heavily depending on geographic region, with frequency rates for Europe ranging between 1.4% and 1.6% [1]. Psoriasis is associated with renal involvement and a current topic for controversy centers on the existence of a distinct “psoriatic nephropathy” entity [2-4]. The objective of this case report is to discuss the current evidence and immunohistological profiles of immunoglobulin (Ig) A nephropathy (IgAN) in psoriasis by presenting a patient with generalized pustular psoriasis (GPP), who presented with rapidly progressive decline in renal function secondary to IgAN. In some case, such as ours, a potential diagnostic challenge may arise when trying to differentiate IgAN from IgA-dominant infection-related glomerulonephritis (IgA-IRGN), another cause of nephritic-nephrotic syndrome, which may present with rapidly progressive decline in renal function after a staphylococcal skin infection.

## Case presentation

A 43-year-old male patient was admitted to our department for rapidly progressive renal failure. One month prior, he was diagnosed with acute GPP (of von Zumbusch). A visit to the doctor was warranted due to a coagulase-negative staphylococcal superinfection, and the diagnosis was subsequently made. At this time, serum creatinine was 0.78 mg/dL.

**Figure 1:**
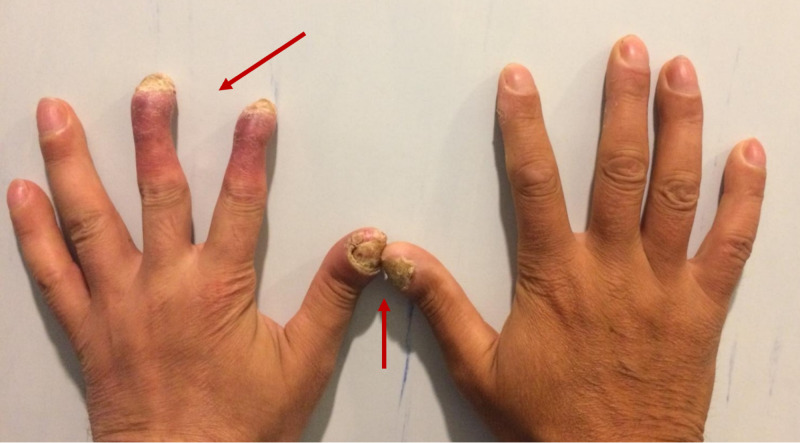
Hand examination showing hyperkeratotic, psoriasiform scaling of digits 2 and 3 on the left hand (arrow) and onychodystrophy of the first digits bilaterally (arrow).

At admission, the patient was afebrile and had multiple erythematous, painful pustules localized to erythematous skin on the abdomen and extremities. Examination of his hands revealed hyperkeratotic, psoriasiform scaling (especially of digits 2 and 3 on the left hand) and onychodystrophy of the first digits bilaterally, similar to findings in acrodermatitis continua of Hallopeau (Figure 1). Mild pitting edema of the lower limbs was present. The patient was dyspneic and had diminished breath sounds over the posterior left basal area. Blood pressure was 150/80 mmHg and heart rate was 64 beats per minute. On abdominal ultrasound, the left and right kidneys were of normal size. Transthoracic echocardiography did not reveal any abnormalities.

Laboratory investigations and their results are shown in Table 1. A moderate normocytic normochromic anemia was present, with elevated acute phase reactants. The patient did not have a history of allergic/atopic reactions to drugs. Viral serology was negative, and multiple electrolyte abnormalities were present. Urinalysis showed pyuria with positive cultures for Klebsiella pneumoniae, while high-grade proteinuria and gross hematuria suggested nephritic-nephrotic syndrome. In light of the rapidly progressive renal insufficiency, biopsy was done to further steer the hospital course. 

**Table 1:** Lab results upon admission. WBC: white blood cell; HBs: hepatitis B surface; HCV: hepatitis C virus; HIV: human immunodeficiency virus; RBC: red blood cell; HPF: high-powered field.

Lab value	Reference range	Lab value at admission
Complete blood count		
Hemoglobin (g/dL)	13-17.5	9.5
WBCs (x10^9^/L)	4-10	15,800
Biochemistry		
Fibrinogen (g/L)	1.8-4	539
C-reactive protein (mg/L)	<5	51
Cholesterol (mg/dL)	<150	175
Triglycerides (mg/dL)	50-150	245
Albumin (g/dL)	3.5-5.0	2.13
Blood glucose (mg/dL)	65-110	90
Alkaline phosphatase (U/L)	50-100	238
Alanine aminotransferase (U/L)	5-30	118
Aspartate aminotransferase (U/L)	5-30	32
Serology		
HBs antigen	Negative	Negative
HCV antibody	Negative	Negative
HIV antibody	Negative	Negative
Urea (mg/dL)	8-21	65
Creatinine (mg/dL)	0.8-1.3	3.22
Uric acid (mg/dL)	4.0-8.5	5.2
Cytoplasmic antineutrophil antibodies (IU/mL)	≤1.9	1.32
Perinuclear antineutrophil cytoplasmic antibodies (IU/mL)	<3.5	0.16
Antinuclear antibodies	Negative	Negative (low titer)
Urinalysis		
Proteins (g/24 h)	≤0.15	3.7
Leucocytes (WBCs/HPF)	≤2-5	10
Erythrocytes (RBCs/HPF)	RBCs - ≤2	230
Urine culture	Negative	Klebsiella pneumoniae

**Figure 2:**
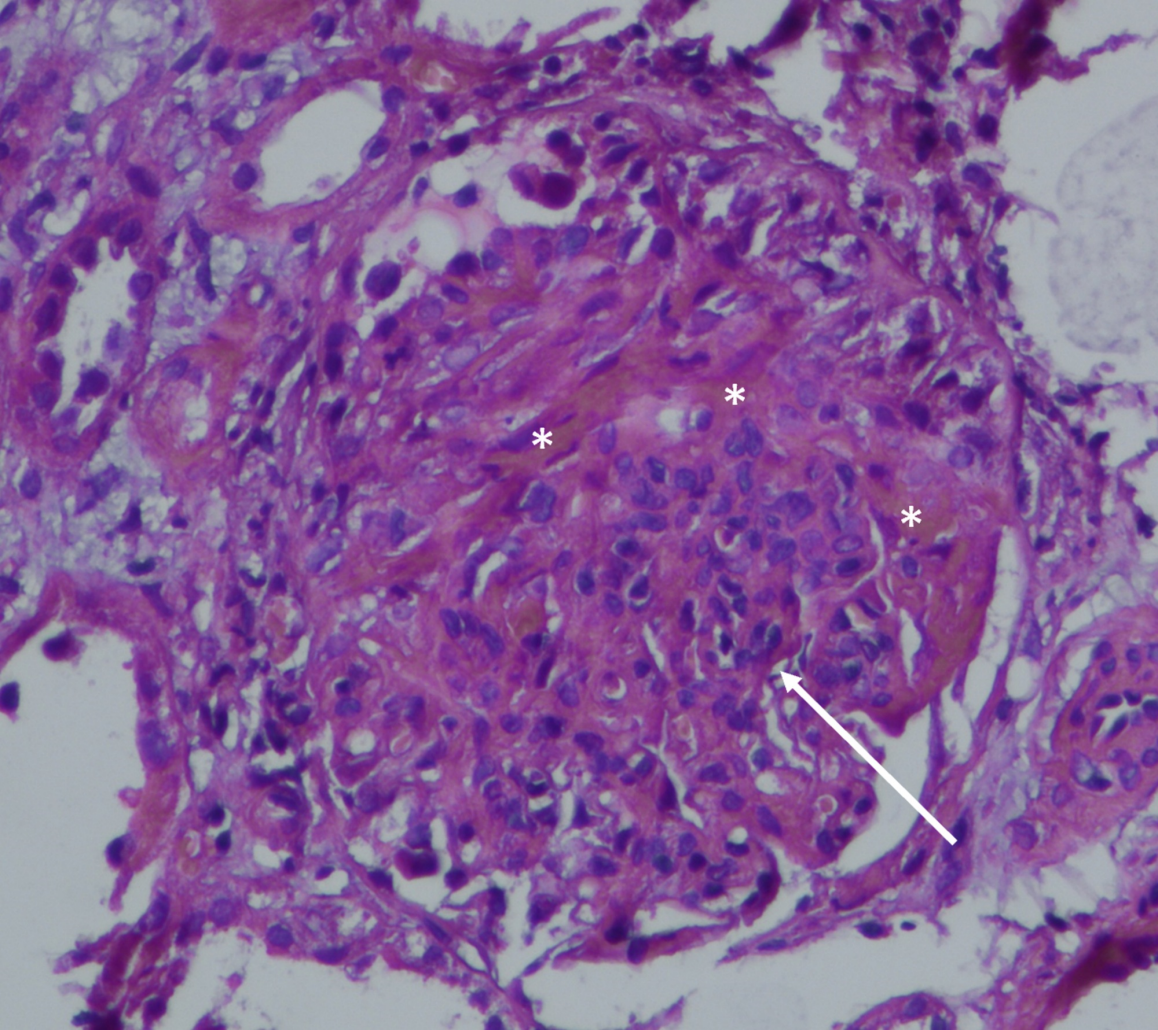
Histologic examination showing a crescent (asterisks) and mesangial hypercellularity with matrix expansion (arrow).

Histologic examination of nine glomeruli showed mesangial hypercellularity in over half of the visualized glomeruli, endocapillary hypercellularity, crescents, and absent glomerulosclerosis or absent tubular atrophy (Figure 2).

**Figure 3:**
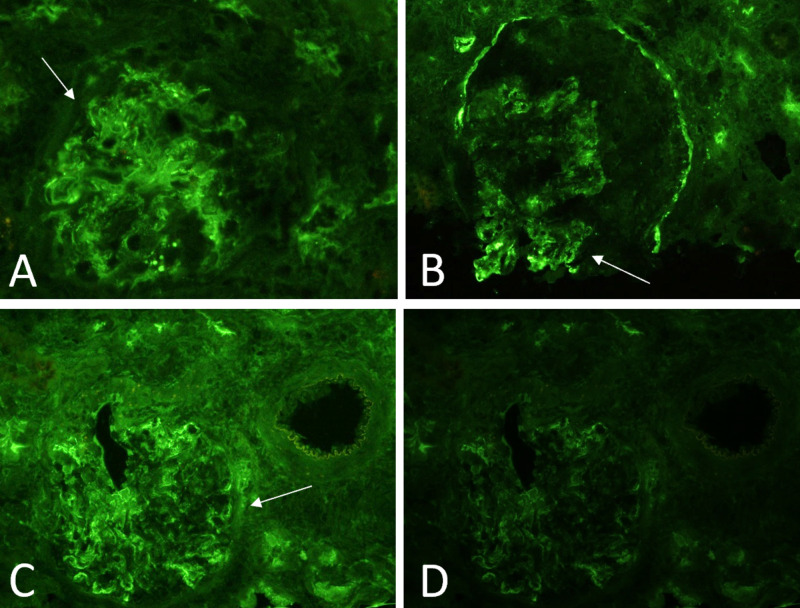
Immunofluorescence showing more intense staining for IgA (A) rather than C3 (B) and larger, more globular staining of lamba (C) than kappa (D) light chains.

Immunofluorescence (IF) microscopy showed extensive mesangial deposition of IgA and C3, in addition to mesangial light chain (λ > κ) positivity (Figure 3).

**Figure 4:**
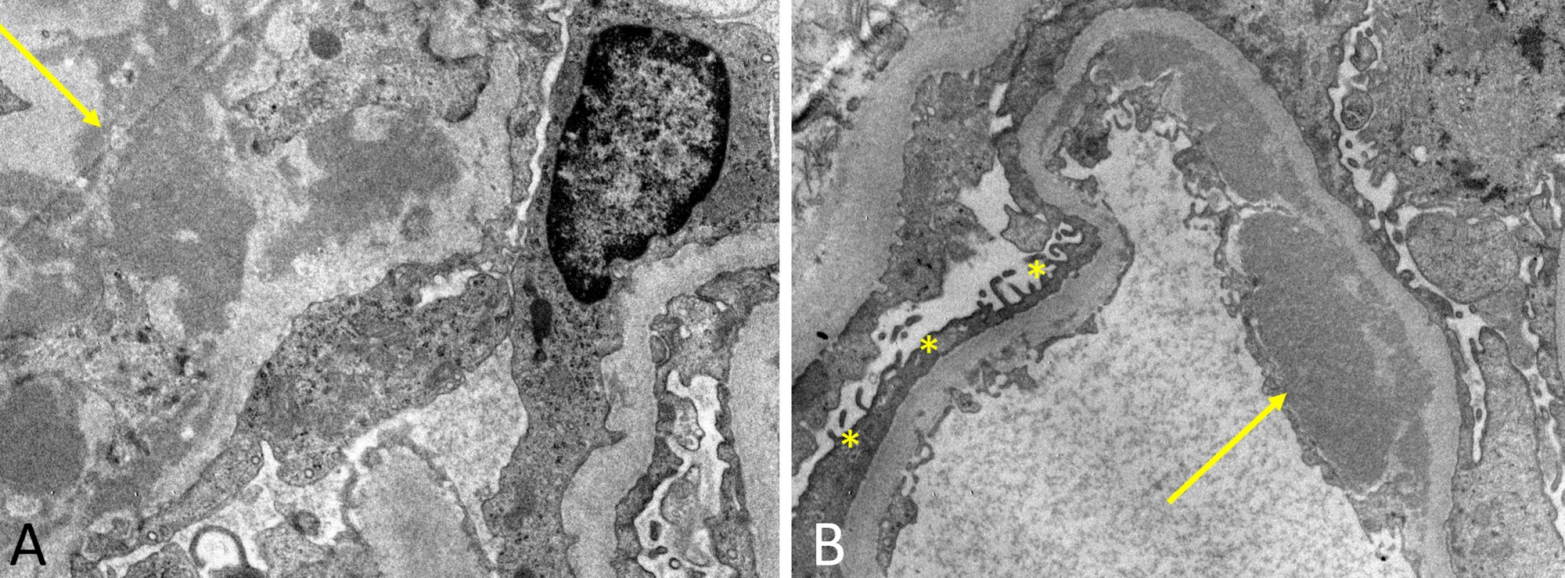
Electron microscopy in image A showing a large electron-dense paramesangial deposit (arrow), without formation of subepithelial humps (thus ruling out post-streptococcal glomerulonephritis) and in image B effacement of podocytes (asterisks) and more electron-dense subendothelial deposits (arrow).

Dense deposits were found on electron microscopy (EM) to be limited to the mesangial regions and were accompanied by endothelial deposits with podocyte effacement (Figure 4). In context of this information, a diagnosis of IgAN was made and an Oxford score of M1E1S0T0C2 was calculated [5].

In the acute setting, pathogenic treatment was started with glucocorticoids (oral prednisone 0.5 mg daily with a progressive decrease in dose after the first month until 10 mg daily) and cyclophosphamide therapy dosed as 0.5 g/m^2^. Prednisone was tapered and discontinued 14 months after it was started. One month later after admission, the patient experienced exacerbation of his psoriasis in the form of erythroderma, which was treated with topical mix of nystatin, retinoids, boric acid, erythromycin, and methylprednisolone.

**Figure 5:**
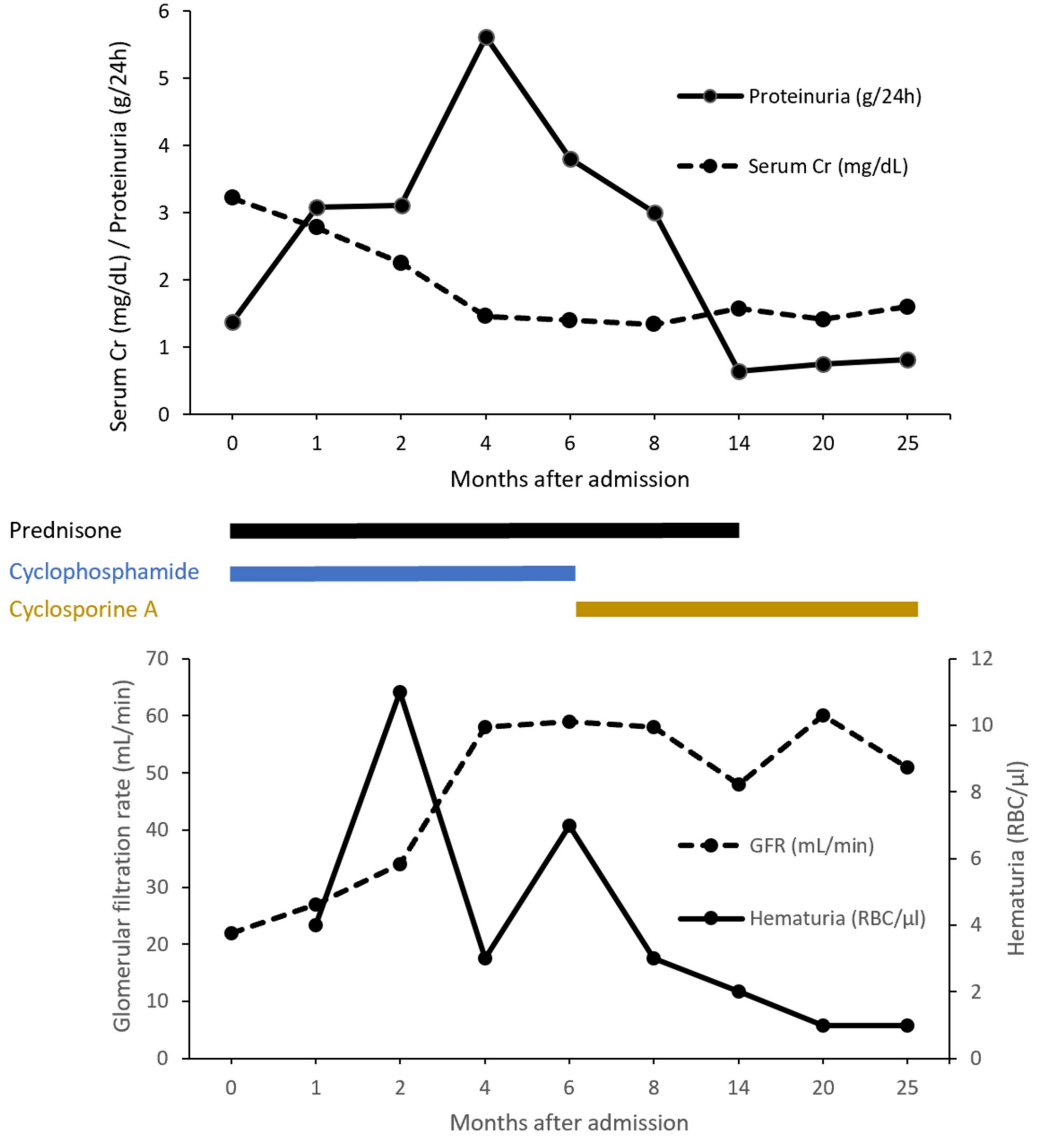
Evolution of the patient's renal function and corresponding treatments over 25 months after initial admission to our department with acute kidney injury. GFR: glomerular filtration rate; Cr: creatinine; RBC: red blood cell.

At discharge, esomeprazole, amlodipine, and cyclophosphamide (administered as pulsed monthly therapy) were added and renal function parameters over the course of 25 months of therapy are shown in Figure 5. Due to the generalized psoriasis and presence of proteinuria and a spike in hematuria values, cyclophosphamide was replaced with cyclosporine A, and the dose was titrated every three months to reach target plasma levels of 100 ng/mL. The evolution of the patient was favorable, with glomerular filtration rate (GFR) stabilizing at approximately 55 mL/min and six months later cyclophosphamide was replaced with cyclosporine. Additionally, a high-intensity statin and vitamin D were prescribed.

## Discussion

We present a patient with GPP who presented with IgAN manifesting as rapidly progressive decline in renal function. There are a variety of clinical forms of psoriasis with GPP, as seen in our patient, being a rare, life-threatening, and severe multisystem form of the disease [6]. Unlike classic psoriasis, the pathoimmunogenic landscape of GPP is still poorly understood, although mutations in genes encoding interleukin-36 antagonists that dysregulate the neutrophil-chemokine axis are believed to play a central role in the disease [7]. The differing pathogenesis and therefore, a lapse in pathobiology of classic psoriasis and GPP also explain why typical therapy with cyclosporine A or anti-tumor necrosis factor biologics rarely achieves complete control of the disease [8]. As a chronic inflammatory systemic disease, psoriasis is associated with an extensive list of comorbid diseases, including renal involvement [9]. Despite the differences in molecular basis, there are nonetheless some similarities and with regards to chronic kidney disease (CKD), the most important factor is severity of the psoriasis rather than subtype.

The association between psoriasis and renal abnormalities has been debated, with authors questioning the role confounding variables, such as hypertension, diabetes, and use of nephrotoxic drugs play in inflating rates of CKD and end-stage renal disease (ESRD) [10]. Although this is a valid argument, it was definitively addressed in the largest meta-analysis to date, where Ungprasert et al. examined 199,808 cases with psoriasis to find a pooled relative risk of 1.29 (95% CI, 1.05-1.60) in favor of higher incidence of CKD and ESRD [3]. Only studies that adjusted the risk of incidence of CKD for the aforementioned confounders were included in the meta-analysis, meaning psoriasis was an independent risk factor for CKD/ESRD and the increased incidence could not be explained by a higher rate of comorbidities or nephrotoxic medications. However, even authors of such a landmark review acknowledged that association does not always mean causation. It is still possible, although unlikely, the relationship between psoriasis and renal involvement is coincidental, as the provisional pathogenesis underlying this phenomenon has been proposed, but not confirmed [11].

Within this context, the most common cause of glomerulonephritis (GN) in psoriasis is IgAN, typically presenting with gradual appearance of proteinuria and hematuria and indolent reduction in estimated glomerular filtration rate (eGFR), unlike our patient [2]. It is also important to mention that the incidence of IgAN in patient with GPP is higher than the general population. The pathogenesis of IgAN begins with production of pathogenic IgA molecules, where aberrantly glycosylated hinge regions (IgA1) reveal amino acid sequences that become targeted by IgG [12]. Ig complexes are formed and deposited in the kidneys during filtration, where they activate complement; for this reason, C3 is often present on IF (as in our patient) and contributes to the severity of the disease. As with most GNs, diagnosis can only be confirmed via biopsy and in the case of IgAN, further classified via the Oxford system to stratify patients according to risk of progression to ESRD [5].

**Figure 6:**
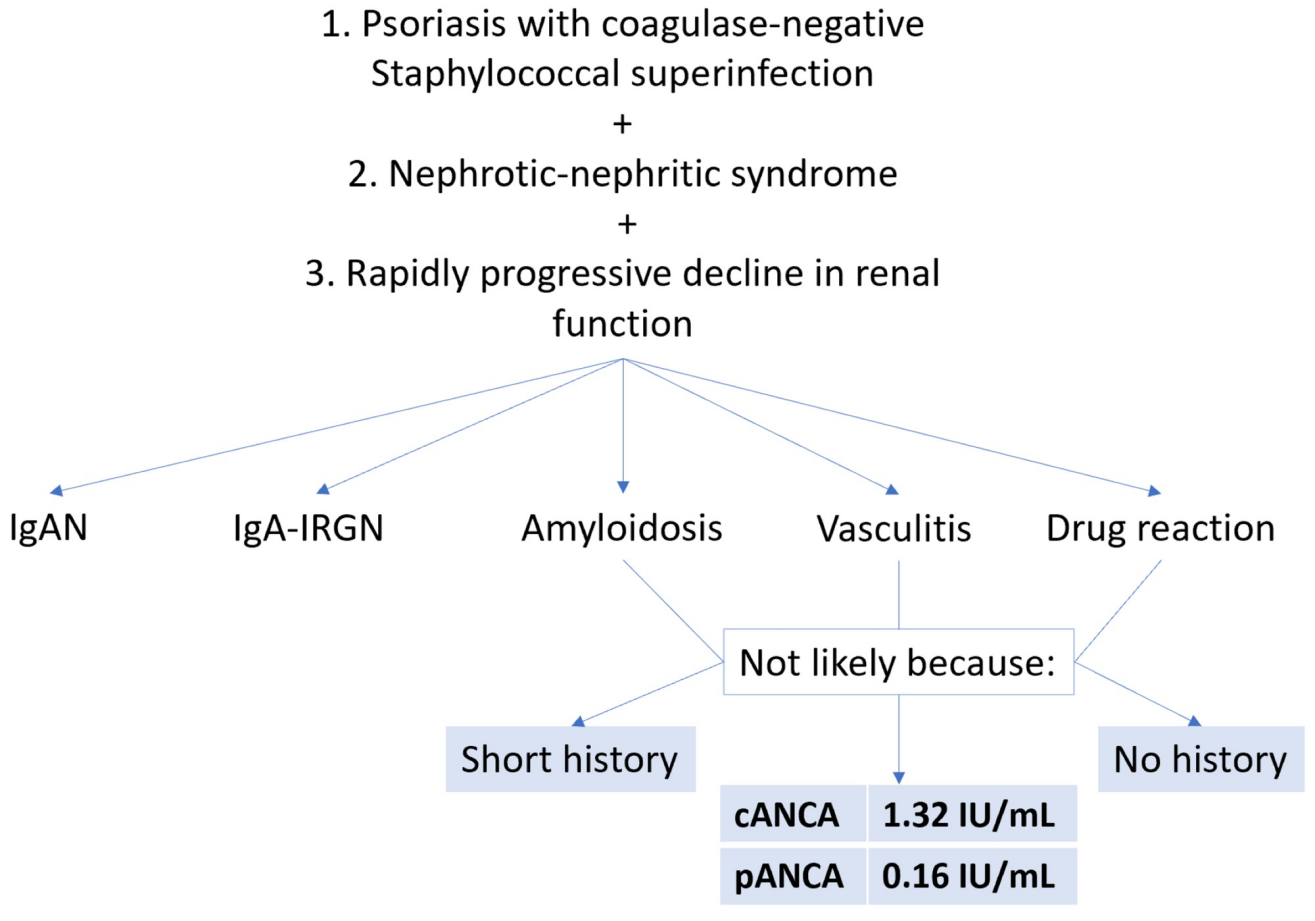
The figure shows our differential diagnoses and reasons for excluding them. IgAN: immunoglobulin A nephropathy; IgA-IRGN: immunoglobulin A-dominant infection-related glomerulonephritis; cANCA: antineutrophil cytoplasmic antibody; pANCA: perinuclear antineutrophil cytoplasmic antibody.

Our rationale during the initial workup and the subsequently encountered diagnostic challenge are presented in Figure 6. Given how the diagnosis of GPP was incited by a coagulase-negative staphylococcal superinfection, the differential diagnosis of IgA-IRGN must be considered. Its incidence has been increasing in recent years, it usually occurs in older patients, and it classically appears after a streptococcal upper respiratory tract infection or a staphylococcal skin infection [13]. Furthermore, the presentation of the renal involvement as rapidly progressive decline in renal function with overt proteinuria was more characteristic of IgA-IRGN [14]. At the same time, in a cohort of 35 patients, the causative agent in the overwhelming majority of cases (94%) was Staphylococcus aureus, with only two cases being attributed to coagulase-negative Staphylococcus epidermidis [13]. This clashing clinical data was another reason to look to the biopsy results for definitive answers.

Handa et al. described the differences in immunohistological profiles of IgA-IRGN and IgAN, and the most significant differences are summarized in Table 2 [15]. In IF, deposition of IgA and C3 along capillary walls is more common of IgA-IRGN than IgAN, where it was largely restricted to the mesangial area. They also found EM findings to mirror IF, finding mesangial dense deposits in IgAN and subepithelial and subendothelial deposits in IgA-IRGN. As such, another case peculiarity we encountered was the presence of crescents in our histologic sample, which complicated diagnosis and required IF/EM to confirm the diagnosis. Unfortunately, due to the rarity of these cases, there is a paucity of high-quality evidence that would detail specific treatment recommendations, prognosis, and how psoriasis affects disease progression.

**Table 2:** Immunohistological profiles of IgA-IRGN and IgAN. IgA: immunoglobulin A; IgA-IRGN: immunoglobulin A-dominant infection-related glomerulonephritis; IgAN: immunoglobulin A nephropathy; LM: light microscopy; IF: immunofluorescence; EM: electron microscopy.

Imaging modality	Immunohistological profile	IgA-IRGN	IgAN
LM	Endocapillary hypercellularity	85%	35%
	Cellular crescents	6.7	2.7
IF	Mesangial IgA	69%	97%
	IgA/C3	↓	↑
	λ/κ	↓	↑
EM	Mesangial dense deposits	46%	93%
	Mesangial hypercellularity	38%	61%
	Podocytes effacement	77%	37%
	Humps	23%	0.8%

Treatment recommendations for IgAN manifesting as acute renal failure include prednisone, cyclophosphamide, and azathioprine, either as eight weeks of induction or maintenance therapy [16]. Per these guidelines, we decided to initiate a combination treatment with glucocorticoids with cyclophosphamide due to the severe renal involvement, rising creatinine, and nephrotic-range proteinuria. At the six-month mark, cyclophosphamide was replaced with cyclosporine A in an attempt to better control renal involvement and the GPP lesions. The role of calcineurin inhibitors in managing proteinuric glomerulonephritides has been debated, in light of considerable concern for nephrotoxicity and the rise in serum creatinine, which was overturned in a recent meta-analysis of 374 patients [17]. Furthermore, cyclosporine A is a first-line treatment for GPP and has been shown to be safe and efficacious treatment [18]. After switching treatments, evolution has been favorable for both the cutaneous lesions and the kidneys (absence of hematuria, protein exceretion <0.5 g/daily, stable renal function). Using the risk-prediction tool based on 13 clinical parameters (including the Oxford score) by Barbour et al., the risk of 50% decline in eGFR or progression to ESRD in the next three years is 27.98% [19]. Monitoring disease activity and perceived progression risk via hematuria, proteinuria, and serum creatinine will be a priority for this patient, and introduce more data regarding renal parameter progression from graph.

## Conclusions

Current evidence confirms the higher incidence of renal involvement in psoriatic patients, as compared to the general population. The predominant form of renal involvement in psoriasis occurs in the form of IgAN, which in rare circumstances may present as rapidly progressive decline in renal function. In instances where an upper respiratory or skin infection has preceded the GN, IgA-IRGN must be considered on the differential. Despite rapidly progressive decline in renal function being more typical of IgA-IRGN, IgAN can still present with this feature and renal biopsy with subsequent IF/EM analysis is the gold standard investigation to differentiate between these two pathologies. Further research is needed to detail specific therapy considerations, prognosis, and how psoriasis affects disease progression.
